# On Vaccine and Variolous Inoculation

**Published:** 1815-04

**Authors:** Robert Kinglake

**Affiliations:** Taunton


### Medical and Physical Journal.

4 OF VOL. XXXIII.] APRIL, 1815. [NO. 194.

"For many fortunate discoveries in medicine, and for the detection of numerous errors, the world is indebted to the rapid circulation of Monthly Journals; and there never existed any work to which the faculty in EUROPE and AMERICA were under deeper obligations than to the Medical and Physical Journal of London, now forming a long, but an invaluable series."—RUSH.

*For the Medical and Physical Journal.*

### *On Vaccine and Variolous Inoculation;* by Dr. KINGLAKE.

I LATELY submitted to the public, through the medium of your Journal, some observations on the apparently prevailing failures of cow-pox as a preventive of small-pox. Since then, increasing rumours and apprehensions on the subject in this neighbourhood have rendered a further consideration of its merits of the utmost importance to domestic tranquillity. When the parents of vaccinated children are alarmed for their doubtful safety against the ravages of small-pox, and when medical practitioners rather join in the consternation by instantly resorting to variolous inoculation for the desired assurance, it is time to examine how the question fairly stands between vaccine security and variolous danger. The vaccine disease has, with some exceptions, very reputably maintained its ground as an effectual preventive of small-pox until very lately. The accidental instances of failure that have occurred, may be referred to either a deteriorated state of the vaccine fluid, owing to its protracted diffusion through the human subject by means of inoculation, by its being taken at a period of the disease when the specific and anti-variolous property of the matter did not exist, or under circumstances of idiosyncratic or accidental conditions of insusceptibility for the disease that rendered its efficacy unavailing for the intended security. Either one or more of those causes may effectually impede the vaccine fluid from exerting its wonted preventive influence. That such cases might present cannot be denied, and that they actually do also occur in variolous inoculation all those who have been most conversant with that practice can readily attest. Why then should casualties be admitted as an insuperable objection to the general course of the disease? to allow of such latitude of objection, would be to argue from an exception rather than from the general rule, a mode of reasoning too illogical to be at all conclusive. A due attention to the causes of the occasional failures will suggest the most probable remedy against them. An uniform reference to the cow for the morbid fluid, generated by the vital actions of its own peculiar animal economy, appears to me, as formerly observed, to be meeting the difficulty *in limine*, and to afford the best chance of completely overcoming it. Neither the preservative efficacy arising from the specifically active powers of the vaccine fluid, nor that of idiosyncratic insusceptibility to its influence, can sufficiently assure the anxious solicitude that ever must exist on the subject.

It is not my desire to assume any thing to the disadvantage of vaccine inoculation, nothing short of unquestionable facts would I admit into the discussion of its merits. The reputed failures are numerous; the real ones, it is my firm persuasion, are but very few; indeed, not more perhaps than may be justly regarded as exceptions to general influence, and to which all powers and properties are more or less liable. The real facts by which the question should be tried would seem to be those which instances of natural, not inoculated, small-pox after vaccination would furnish. But two cases are, at present, distinctly in my recollection of such occurrences, one of which appeared to be decisive of vaccine inefficacy as a preventive, the other was too doubtful to be adduced as an authority, it, therefore, fell short of the character which real facts ought to possess on the occasion. I have, indeed, heard of many others, but no opportunity has been afforded me for verifying them.

The natural small-pox is too vague a phrase to decide on the reality of that affection. This disease may occur under different circumstances, having an apparent sameness of character, but radically dissimilar with respect to the constitutional and local nature of the disease. The distinction arises from the previous as well as concomitant symptoms. When shivering, sickness, head-ach, and prostration of strength, succeeded by febrile reaction terminating in eruptions on the cutaneous surface, and running the usual course of small-pox, there can be no question of the real nature of the disease; but, when similar eruptions on the skin shew themselves unpreceded and unattended with the febrile character of constitutional small-pox, it is by no means conclusive that such a disease is any thing more than a local affection of the skin, such as may happen again and again in any person who may have previously undergone either the natural or inoculated small-pox. That such instances of merely cutaneous small-pox may occur after vaccination, is no more than what equally happens after the small-pox itself, and and cannot be admitted as justly impugning the anti-variolous power of vaccine disease.

The instances with which the medical world begins to abound of small-pox being imparted to persons who had previously been subjected to vaccination merit, I am firmly of opinion, to be for the most part considered as sheerly cutaneous affections, and do not warrant any thing like a conclusion against the protecting efficacy of vaccine inoculation. Where is the skin that will not be more or less irritated by inserting under it variolous matter? and, if it be inevitably irritated in a degree, what is to hinder an extension of that effect to the length of inducing eruptions over the whole surface. At the place of inoculation, a variolous action is excited, which may either exhaust itself on the part, or become diffused over the whole skin, according to circumstances of contiguous and remote sympathy, and to the greater or less irritability of the skin. It will not, then, be just to denominate an inflamed arm from variolous inoculation, whether attended or not with eruptions, a case of real small-pox, provided it be unaccompanied with the usual symptoms of variolous disease. It is not enough even that nausea, head-ach, and an increased action of the heart and arteries, should follow the local stimulus occasioned by inoculated small-pox, when it quickly ensues its insertion; it is then no more than simple excitement, if the system at large should become insusceptible of variolous contagion from either having before undergone the disease or rendered inaccessible to it by vaccination. A striking instance of this kind has just occurred in my own family: A son of mine, aged about seven years, was lately the subject of singular irritation from the insertion of variolous matter. Within twenty-four hours, the inoculated part of the arm became violently inflamed and swollen, with a suppurated point of the size of a large small-pox eruption; this high excitement induced nausea, vomiting, head-ach, sore throat, extreme rapidity of pulse, coated tongue, and a general efflorescence over the skin. The arm remained equally inflamed until the second day, and until then the suppurative process continued; at about that time, the local irritation diminished, and, with that abatement, the symptoms of general disease also subsided. The local inflammation then rapidly declined, leaving, on the eighth day, an incrusted cicatrix considerably elevated above the level of the surrounding skin, similar to the protuberant eschar usually remaining after a successful inoculation for the small-pox. An areola was also apparent around the inflamed part until that time; an eruption, likewise,

 near near the inoculated sore appeared on the fifth day, which also began to retreat on the eighth day.

The inordinate course assumed by this experimental inoculation induced a momentary anxiety in my mind, that parents only can appreciate, and which no suggestion of medical philosophy could wholly repress. The event, however, (as might have been justly anticipated) proves it to have been a case of local irritation, going the length of involving the system by the strong sympathetic influence it excited. The progress and termination of the symptoms clear the case of all suspicion of its being variolous, and prove that the system has been, as was confidently expected, effectually guarded by the vaccination to which it had been five years before subjected.

The recording of this case may serve the practical good of furnishing a caution not unnecessarily to expose children to the possible effects of variolous inoculation, for who can with certainty say, that, however the variolous infection may be rebutted by the system generally, that it may not induce very serious, nay, mortal mischief on the part where the morbid poison may be inserted. That this may be the case, I suspect is all but fatally proved in the instance under consideration. If the inflammatory action had spread erysipelatously over the skin, instead of sinking into the muscular structure in a phlegmonous manner, it is conceivable, and to my feelings, circumstanced as I was, it is horrible to reflect on it, that, instead of ending in suppuration, it would have terminated the life of the dear little sufferer by gangrene. Since the fifth day of the disease, the time when the above remarks were written, the little patient remained apparently free from all complaint until the morning of the ninth day, when sickness, vomiting, and head-ach, attended with quickened pulse, heated skin, coated tongue, and great thirst, again occurred. Repeated and copious vomiting relieved the pressure of disease at the time, but the symptoms recurred on the following morning, the 11th of the disease, with increased violence, and again were removed by plentiful vomiting. On the 12th day of the disease, slight head-ach existed, with a tendency to nausea. The diet was confined to a small quantity of vegetable substances, and no fermented liquor was allowed, the bowels were sufficiently open, and in two or three instances freely moved by medicine. The state of the child at that time was as might be expected in the ordinary course of infection from variolous inoculation, yet not the slightest variolous action appeared to be going on at the inoculated part, nor was there a smallpox pox eruption on any part of the surface. May not the vaccine influence, where it does not altogether resist the action of small-pox, so modify its character as to induce the inordinate appearances that have been exhibited in this case, and is it not a perilous adventure to bring into collision the irregular actions of too different diseases? Is it not imposing on the healthy energies of life, a conflict of doubtful and dangerous issue?—These questions are intended as seasonable checks at this period of ungrounded alarm against the prevailing disposition to fly to the insertion of variolous matter, as the only safeguard against the uncertainty of vaccine efficacy. It cannot reasonably be supposed,—all analogy indeed is against the supposition,—that a morbific fluid, so virulent as that of small-pox, can have ingress to the system with impunity. It may prove innoxious, but the chances of mischief would seem to have a fearful odds against its being altogether inoffensive.

I am strongly of opinion, that were persons, who have undergone either the natural or inoculated small-pox, to be subjected to the action of variolous matter, as it is commonly inserted in those who have been vaccinated with a view to be decidedly secure from small-pox, that in most instances effects would be produced similar to those that arise after vaccination; that is to say, that the inoculated part would irregularly inflame, and that, on some occasions, eruptions on the skin might appear, and that symptoms of fever might occur, according to the state of irritability in which the trial may find the skin and the system at large. It is time that the perplexities in the public mind on this subject should be explained and removed, and that the point in question should be investigated by a patience of research and a clearness of enquiry far exceeding any thing that has been hitherto shewn on the occasion; then, and not till then, will information be afforded that may serve as a steady and an infallible guide amidst the various and contradictory notions of an undiscriminating public, and the pusillanimous and vacillating demeanour of some of the medical faculty.

*Taunton;* ROBERT KINGLAKE.

*Jan.* 26, 1815.

---

*For the Medical and Physical Journal.*

